# Multiscale Innovative Materials and Structures (MIMS)

**DOI:** 10.3390/nano12010096

**Published:** 2021-12-29

**Authors:** Raffaele Barretta, Domenico De Tommasi, Fernando Fraternali

**Affiliations:** 1 Department of Structures for Engineering and Architecture, University of Naples Federico II, Via Claudio 21, 80125 Naples, Italy; 2 Department of Civil, Environmental, Land, Construction and Chemistry, Polytechnic University of Bari, Via Edoardo Orabona 4, 70125 Bari, Italy; 3 Department of Civil Engineering, University of Salerno, Via Giovanni Paolo II, 132, 84084 Fisciano, Italy

Increasing attention is growing towards advanced multiscale metamaterials and nanostructures, due to recent developments in nanoscience and nanotechnology. Smart nanoscale systems with unconventional features are attractive subjects for many applications, such as electronic, optical and magnetic devices, involved in many areas such as aerospace and mechanical engineering, medicine, biology and sound and heat control. The modeling, characterization and synthesis of innovative materials and structures are, thus, needed to explore new challenging scientific applications.

The Special Issue (SI) Multiscale Innovative Materials and Structures provides new insights concerning the modeling and realization of advanced nano-reinforced materials, carbon nanotube-based systems, tensegrity structures, periodic lattices and multiscale composites. Theoretical and experimental outcomes collected in the SI provide original and significant contributions in the field of nano-composites, nano-systems and nano-devices.

The first work of the SI [1] deals with the functionalization of multi-walled carbon nanotubes, showing the effects of different conditions and milling processes on thermal and electrical conductivity. Another challenging topic regarding advanced sensing applications is explored in [2], where hybrid carbon nanotubes are employed to develop a self-sensing cementitious stabilized sand by virtue of the piezo-resistivity principle. Advancements in nonlocal continuum field theories are given in [3], where an effective model of a nonlocal foundation is presented, providing an accurate and well-posed displacement-driven formulation in modeling soil–structure interactions. New developments in the framework of non-Newtonian rheology are provided in [4]. Notably, a multi-relaxation time lattice Boltzmann scheme coupled with an immersed boundary technique is adopted to describe the evolution of a non-Newtonian fluid. In the modeling of advanced materials, a growing interest is aiming towards nanocomposites due to their special features such as strength and toughness. Fiber-reinforced concrete enhanced with nanofillers is investigated in [5], where the structural response is predicted by adopting a diffuse cohesive interface approach. The potentiality of tensegrity structures for the optimal design of next-generation biomimetic fibers is investigated in [6], where a tensegrity model of spider dragline silk is established. The model can efficiently reproduce phenomena such as the Poisson effect, a tensile stress–strain response and fiber toughness. Exceptional properties of cellulose nanofiber are extensively studied in [7], enlightening the effects of the use of citric acid for hydrothermal carbonization. The effects of acetone rinsing on the thermal stability and surface area of hydrochars are also investigated. New insights on the modeling and fabrication of tensegrity metamaterials are provided in [8], where unconventional static and dynamical mechanical behaviors of bistable lattices with a tensegrity architecture and nanoscale features are deeply examined. The last paper of the SI [9] is a comprehensive review about nanocrystalline soft magnetic iron-based materials based on Fe–Si–B doped with various chemical elements. All steps from realization to application are explored with an in-depth analysis of physical processes occurring at different stages.

It is our hope that the works collected in Multiscale Innovative Materials and Structures will provide scientific community with novel and significant contributions to the topic.
